# Impact of research activity on performance of general practices: a qualitative study

**DOI:** 10.3399/BJGPO.2024.0073

**Published:** 2024-09-04

**Authors:** Cassandra Kenning, Juliet A Usher-Smith, James Jamison, Jennifer Jones, Annette Boaz, Paul Little, Christian Mallen, Peter Bower, Sophie Park

**Affiliations:** 1 Centre for Primary Care and Health Services Research, The University of Manchester, Manchester, UK; 2 The Primary Care Unit, Department of Public Health and Primary Care, University of Cambridge, Cambridge, UK; 3 Division of Psychiatry, University College London, London, UK; 4 Department of Population Health Sciences, University of Leicester, Leicester, UK; 5 NIHR Health and Social Care Workforce Research Unit, Policy Institute, King’s College London, London, UK; 6 Primary Care Research Centre, University of Southampton, Southampton, UK; 7 Keele University, School of Medicine, Keele, UK; 8 Research Department of Primary Care and Population Health, University College London, Hampstead, UK

**Keywords:** research activity, general practice, qualitative research, research

## Abstract

**Background:**

There is evidence that engaging in research is directly associated with better performance. If this relationship is to be strengthened, it is necessary to understand the mechanisms that might underlie that relationship.

**Aim:**

To explore the perspectives of staff and wider stakeholders about mechanisms by which research activity may impact on the performance of general practices.

**Design & setting:**

Qualitative study using semi-structured interviews with general practice professionals and wider stakeholders in England.

**Method:**

Individual interviews with 41 purposively sampled staff in ‘research-ready’ or ‘research-active’ general practices, and 21 other stakeholders. Interviews were independently coded by three researchers using a framework approach.

**Results:**

Participants described potential ‘direct’ and ‘indirect’ impacts on their work. ‘Direct’ impacts included improved knowledge and skills that could change practice work (for example, additional records searches for particular conditions); bringing in additional resources (for example, access to investigations or staff); and improving relationships with patients. ‘Indirect’ impacts included job satisfaction (for example, perception of practice as a centre of excellence and innovation, and the variety afforded by research activity reducing burnout); and staff recruitment (increasing the attractiveness of the practice as a place to work). Responders identified few negative impacts.

**Conclusion:**

Staff and stakeholders identified a range of potential impacts of research activity on practice performance, with impacts on their working lives most salient. Negative impacts were not generally raised. Nevertheless, responders generally discussed potential impacts rather than providing specific examples of those impacts. This may reflect the type of research activity conducted in general practice, often led by external collaborators.

## How this fits in

Research usually improves outcomes through translation into practice, but there is developing evidence that research activity itself may improve the performance of healthcare organisations. However, the bulk of the existing evidence relates to hospital settings. We interviewed general practice staff and other stakeholders to understand how taking part in research might improve practice performance. They identified a range of potential impacts of research activity on practice performance. Impacts on their job satisfaction and working lives were most salient.

## Introduction

Although the benefits of research are traditionally thought to occur through its implementation into practice,^1^ there is increasing evidence that engaging in research activity itself is directly associated with better performance.^2–7^ When clinicians and health organisations are engaged in research, improvements have been shown in health services performance, patient health outcomes and improved processes of care. For example, hospitals with high levels of cancer research also show better outcomes even among their patient populations with cancer who are not directly participating in research.^3^ Most of this evidence is from secondary care, but there is a developing literature from general practice also suggesting associations between research activity in a practice and improved performance by that practice on a range of measures.^8–12^

If such a relationship exists, an important question concerns the mechanisms that might underlie that relationship, as an understanding of mechanisms is necessary if the relationship is to be strengthened and supported.^13^

A published review identified five potential mechanisms linking research activity and outcomes.^9^ Mechanisms included ‘absorptive capacity’, where research activity leads to changes in the ability of organisations to use information effectively, and specific ‘improvements in care processes’ through research (such as greater monitoring of research patients). Other potential pathways included using research to identify problems in organisational processes, or changes arising through greater links between organisations through the research process (such as between practice and academic teams or research network staff). The review also distinguished ‘intentional’ impacts of research activity from those that reflected an indirect ‘spillover’^14^ or ‘ripple’ effect.^15^ Additionally, there are impacts that were specific to particular research studies (that is, a trial in a single clinical condition raising quality of care in that condition) to impacts that are not restricted to that research area.

This initial categorisation of mechanisms drew on a literature dominated by hospital studies and reflected a range of international healthcare systems. The authors highlighted the need ‘*to build understanding of mechanisms, and to explore potentially negative impacts of research engagement alongside benefits*’. Such exploration is particularly needed in general practice, where there is limited literature available.^16^ General practices are smaller organisations, serving local, diverse patient populations, with potentially different activity outcomes compared with hospitals. Research activity in general practice is varied in scope, ranging from large trials to qualitative studies. It includes interventional and observational research; primary and secondary data (that is, research databases such as the Clinical Practice Research Datalink); and publicly funded and commercial research. Research activity also varies by amount (for example, numbers of studies and patients), duration, and complexity; the scale of clinician, practice team, and patient involvement; and the level of support from industry, academic, and research network partners. Finally, research might be more or less focused on questions related to routine general practice clinical work, as only a minority of studies may derive directly from the experiences and needs of general practice staff and their patients.

In this study, conducted in collaboration with a large and cross-England Patient and Public Involvement (PPI) group, we explored the views of general practice staff and wider stakeholders on the mechanisms by which engaging in research might impact on practice performance.

## Method

### Sampling

Twenty general practices were sampled for variation based on publicly available data on their size, location, patient demographics, and quality of care, combined with data on research activity and outcomes from the National Institute for Health and Care Research (NIHR) Clinical Research Network and contextual information gathered through liaison with local Clinical Research Network (LCRN) staff. Based on discussions with PPI contributors, we included practices that had recently been through a process of becoming ‘research active’ through their LCRN and those that were considered highly ‘research active’. To become ‘research active’, practices go through a process of registering with the LCRN and completing the Good Clinical Practice (GCP) training before starting any research activity. ‘Non-research active’ practices had not engaged with their LCRN or expressed interest in becoming research active. We found year-on-year research activity very variable, with some practices increasing activity and some decreasing at the time of interview.

### Recruitment

An information sheet was distributed through local networks and direct mailings to practices. Clinical and non-clinical staff in the practice who had a role or held an interest in research were informed about the study. We also recruited stakeholders from agencies involved with research in general practice, such as research networks and local primary care organisations, based on recommendations from practice staff. For ethical approval, we set a limit of 100 participants, although this was designed to be flexible in case some interviews were done with multiple participants.

### Data collection

Data were collected via semi-structured interviews either in person or remotely (Microsoft Teams or Zoom) lasting between 30 minutes and 60 minutes, between 2 December 2021 and 28 September 2022, by three local researchers CK (female; University of Manchester), JJones (female; University College London), and JJamison (male; University of Cambridge). All interviews were conducted individually. Written or verbal consent was sought at interview. We recorded age, sex, ethnicity, as well as current employment, role, and job satisfaction and each participant was given an identification code.

Interview schedules were broad, including experiences of research, characteristics of an ‘effective’ research practice, and the management of overlap between clinical and research activity, although the analysis presented here is focused on staff perceptions of mechanisms linking research activity and outcomes.

Interviews were audio-recorded, transcribed professionally, and imported into NVivo (version 12) for analysis. Field notes were also collected to better understand practice organisation, culture, and their wider context.

### Analysis

Framework analysis^17^ was used with the support of NVivo (version 12). All researchers went through a process of familiarisation with the first four interviews. Each transcript was carefully read, and a code applied to describe or interpret passages. This stage of open coding was completed independently by the three researchers. A coding frame was then developed by consensus. The working analytical framework was then applied independently by the researchers to transcripts of the interviews they conducted. Framework matrices were generated through NVivo. The research team met regularly in ‘data clinics’ to discuss emerging findings and update the interview schedules as needed. Once coding was complete, the separate NVivo files in London and Cambridge were sent to Manchester for further analysis and the development of themes across the dataset. Two PPI events were held to explore their views on the emergent themes and incorporate their feedback.

## Results

In total, 62 interviews were completed (Table 1). Around two-thirds of practice staff participants were female, ages ranged between 22 years and 56 years, 41% worked full-time at the practice, and the majority reported high levels of job satisfaction (Table 2).

**Table 1.** Interview participants by role

Role	Frequency
**Total practice staff**	41
GP	17
GP trainee	3
Nurse	8
Practice manager	5
Trials coordinator	3
Administrator	3
Pharmacist	2
**Total stakeholders**	**21**
Primary care network	4
Clinical commissioning group^a^	4
NIHR Clinical Research Network	8
Other	5

a Now known as 'integrated care systems'.

**Table 2.** General practice staff characteristics

Variable	Staff (*n* = 41)
Female (%)	28 (68%)
Age years, *n* (%)	
21–30	4 (10)
31–40	17 (41)
41–50	12 (29)
51–60	7 (17)
White British (%)	27 (66%)
Years in role, *n* (%)	
<5	18 (44)
6–15	12 (29)
>15	9 (22)
Full-time, *n* (%)	17 (41)
Job satisfaction score (1–7 scale), *n* (%)	
High satisfaction (5–7)	34 (83)
Low satisfaction (1–4)	6 (15)

### Mechanisms linking research activity and general practice performance

**Figure 1.**
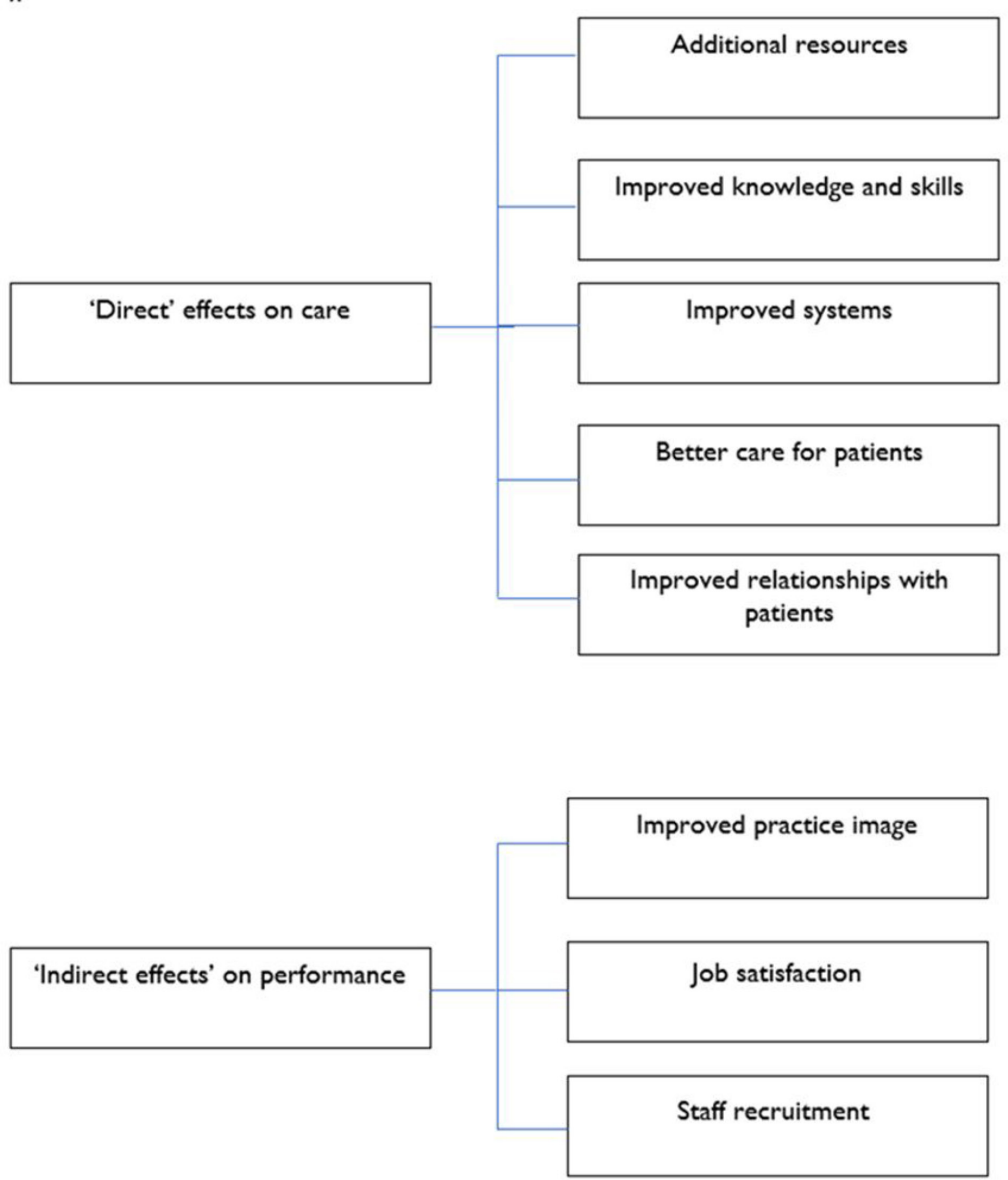
Mechanisms linking research activity and general practice performance

We extracted the themes relating to mechanisms, which we divided into ‘direct’ mechanisms (where there was a clear link between the mechanism and general practice performance) and ‘indirect’ mechanisms (where the link was mediated through broader changes). The core themes are presented in Figure 1 and further expanded in the text.

### Direct effects on care

#### Additional resources

One of the main benefits of research activity was the access to extra resources, including extra income that could be reinvested into the practice:

*'And then secondly we also try to use research as a way to boost our revenue a little bit, so we do try to have a nice mix between commercial studies and ones that benefit the patients, obviously the ones that can help generate some revenue for us is always good, particularly ones where we can afford to fund more staff to get involved with the research projects. Because we want to keep growing and I think the way we see doing that is by creating a big enough revenue stream that we can almost have a research team embedded in the practice*.' (M03S04-Practice Manager)

Some studies were seen as beneficial because they provided access to equipment such as scanners, centrifuges, freezers, or home-monitoring devices. Not all of the benefits described were clinical. Staff and stakeholders described the value of time for patients, with increased appointment times for recruitment and access to staff (such as research nurses) giving patients someone else with time to attend to them, again relieving pressure on practices:

*'a lot of our patients, they enjoy … we don’t have the time restraints that you would have with general practice. So, we can see a patient for up to an hour, whereas in general practice, maybe you are limited to a sort of seven-minute time slot, or a ten-minute time slot. And they also get, almost like a backdoor into the surgery*' (CS407-Clinical Trials Manager)

#### Improved knowledge and skills

Taking part in research was seen by some practitioners as a way of keeping up to date with innovations in health and care, including awareness of new treatments. Trials were seen as a way of potentially ‘upskilling’ the practice team by providing extra learning about new procedures. For those who were involved in the set-up of studies, reading study protocols could provide an update on current evidence for particular conditions:

'*Well, I think every time we have a new research project, and we’re all talking about them and, especially with my team, there’s new syndromes that they never even knew existed, or new words or new ways of seeing people or new thought process, of how can we help with this in a different way? So, for example, the inflammation markers was an interesting one, so we were all talking about that. Oh my goodness, because we’ve got people who we know, who suffer from depression, but there might be an inflammatory cause behind it. So yes, it creates conversation and interest, yes*.' (CS508-Care Coordinator)

#### Improved systems

General practice staff are often involved in the identification of potentially eligible research participants. Responders noted that the processes of running searches and identifying eligible patients gave them the opportunity to maximise accuracy and consistency of coding, helping to improve care through maintenance of systems for identification and follow-up of patients. This had the further potential for improving assessment of practice performance in the Quality and Outcomes Framework pay-for-performance scheme, and the external ratings of practices by the independent regulator (the Care Quality Commission):

*'And sometimes it can be, from our point of view, being able to flag up and pick up patients a lot better after sometimes the research team have done searches on the practice numbers and recognise that actually there are these patients that haven’t been coded, for example.*' (MSH05-GP partner and Research Lead for PCN)

#### Better care for patients

Some practitioners thought that taking part in research could give them access to more modern and evidenced-based services, such as increased access to specialist services (for example, mental health) or ‘extra’ tests for their patients, facilitating access for patients, and helping relieve burden on practice resources:

*'So usually one of the first things we look at is benefit for the patients, so we look at opportunities that patients might not have access to elsewhere. We’re from quite a deprived area and there’s obviously quite a lot of disparity in health care of quite poor outcomes, so we tend to look for studies that have good outcomes for patients first of all.'* (M03S04-Practice Manager)

Another impact on patient care raised was that screening patients for study participation might lead to reviews of specific patient groups and picking up on clinical issues that may have otherwise been missed or detected later. While this potentially could lead to increased workload for practitioners, practices perceived this as a potential benefit for patients. For example, studies around chronic kidney disease (CKD) where patients were told that they have CKD following a search of practice records, reportedly generated additional work in terms of patient queries and appointments to explain the diagnosis to patients who were not aware they had it. However, this ‘extra’ work was recognised as being in line with best practice:

*'I think although in that case it was a little bit more work, actually it highlighted something that we should have been doing anyway. He should have known that he had kidney disease and someone should have had that conversation. I expect there will be a few more bits like that that will probably bring us in to line with best practice as well. So it might be a little bit more work but I think it is all in the right direction. It has not deviated from what we are doing in terms of patient care on a day-to-day basis and it fits in with managing his CKD.*' (M04S07-GP, salaried)

#### Improved relationships with patients

In research-active practices, some staff reported that patients may view staff as more knowledgeable, taking more notice of advice they give. Some practitioners reported that engaging a patient in research gave them the opportunity to build their relationship and increase trust. Other members of practice staff also saw these benefits, reporting more positive feedback from patients involved in research:

'*I think like a lot of other practices, we struggle with some negative feedback at times with how hard it is to meet demand and everyone wanting appointments and everything the same day or exactly when they want, so you can sometimes get in a bit of a negative environment with that, but this as a separate arm, the patients have all been fantastic when they’ve been on these studies. So I think those ones that have been involved so far have seen a massive positive benefit and we’ve noticed the positive feedback which is something we don’t always get.*' (M03S04-Practice Manager)

### Indirect effects on performance

#### Improved practice image

Although responders reported that improved relationships with patients could result from their active participation in research, others spoke of the wider impact where being known for taking part in research had a positive effect on the practice reputation, with these practices being thought of as more ‘progressive’ and ‘innovative’ compared with others. Stakeholders involved in research outside the specific practices also suggested that research activity was indicative of a well-run practice that must be doing the everyday clinical work effectively to be able to take on additional tasks.

#### Job satisfaction

One of the main benefits reported by staff active in research was the positive impact on their job satisfaction. In a system under pressure, the variety offered by research within their daily routine was seen as essential in helping maintain a good-working environment. Particularly, GPs reported that research activity complemented their clinical roles, offered variety, revitalised them during their clinics and therefore potentially reduced burnout:

*'Other reasons, so from a selfish point of view, it just varies the week for me. I think when you do slightly different things it keeps you fresh mentally, does keep you going. And if you do … I think if … if you do the same thing again, and again, especially in GP, you can burn out quite quickly.*' (M03S05-GP partner)

Other factors raised included improved self-esteem, taking pride in research work and making a contribution to national and global health. A further theme from some of the practice staff interviews was improved teamworking owing to delivering research, in particular across practices when research involved activity across a wider primary care network:

*'I think so, and I think the other thing about general practice often in this job is, you work in silo, so when you’re doing research studies it actually … it’s an opportunity to work in very small groups with other people and deliver something where you’re all working on the same project as others. So, it’s nice having these little mini projects to work on, gives a focus for the group, and actually helps engagement within teams as well*.' (L01S01-GP partner)

#### Staff recruitment

Research activity of general practices was not always explicit to all of those responders in the practice, and indeed not always visible outside of the practice. However, some interviewees did report that it could influence recruitment and that they thought this would increasingly become a ‘*selling point*’ in the future:

*'Yes, I think it’s something that’s advertised, you know, when we’re recruiting for staff. It’s something that interested me in coming back to the practice so I’ve spoken to other people who have been thinking about applying for jobs here and it is something that people are interested in, so yes. … I don’t know about the wider staff in general. For me, it makes me more inclined to stay here because, you know, it’s something that not a lot of GP practices do. But yes, I don’t know about everybody else.*' (CS311-GP, salaried)

There were also comments on the positive effects being a research-active practice might have on the type of staff attracted to posts. For example, people identified as ‘*forward-thinking staff*’ and those who are progressive or keen to learn by offering expanded roles or portfolio careers.

## Discussion

### Summary

Among a range of staff in practices demonstrating a range of research activity, multiple potential impacts of research activity on practice performance were identified (see Figure 1). Of those discussed, the impacts on their working lives were most salient.

### Strengths and limitations

Our recruitment procedures ensured a reasonable level of geographical diversity and we were able to use a range of data to sample, according to levels of research activity. Nevertheless, although we achieved good variation in some characteristics, it is likely that volunteers for the study would have represented more research-active practices (by design) and those likely to have had a more positive experience of research activity, and who may have been more willing to entertain the idea that such research improved practice performance. Although it is possible that these impacts would motivate future research activity, our focus was not on why practices engaged in research, but the wider impacts they reported when they did. We interviewed wider stakeholders as we felt that such staff would have a complementary perspective on the benefits of research across a range of practices compared with practice staff working within a single practice. It should be noted that research activity is integrated into care quality inspections in hospital settings, but not primary care.

Framework analysis allows a systematic and flexible approach to coding qualitative data. Using a framework approach ensured consistency in coding across the three sites (Manchester, London, and Cambridge) and allowed us to explore patterns and differences. Regular meetings and discussion of codes resulted in changes to the framework and interview schedules to focus on areas of interest that arose in particular interviews. We did not take an explicit realist approach although further analyses could explore the mechanisms we outline in this article and the contexts that may make them more or less important, which could include characteristics of staff, the research activity undertaken, and the wider practice population or context (such as region or levels of deprivation).

This research was conducted at the end of 2021 to mid-2022, after most COVID-19 restrictions had been lifted, although the impacts were still evident. Many of the practices still limited waiting in patient areas, were using remote consultations, and reported reduced capacity for ‘extras’ such as research activity, or a focus on COVID-19-specific research.^18^

### Comparison with existing literature

As noted previously, the categorisation of mechanisms developed by a previous review^9^ was developed from a broader literature. We mapped our data to those categories and found some similarities, including some evidence of increases in ‘absorptive capacity’ and ‘improvements in care processes’. The former saw impacts such as additional resources as a result of research, reinvestment of research income, training, and updated knowledge of treatment options. Improvements in care processes included access to care, such as a treatment patients would not usually be able to access, which is a fairly common finding in patients;^19^ increased monitoring through trial follow-up visits; and extended appointments. There was less reporting of the other mechanisms. Some responders reported impacts related to the mechanism ‘organisational mechanisms within healthcare system’, such as research processes identifying issues (such as poor coding). However, this was relatively uncommon. Formal collaborative linkages driven by research were also uncommon (beyond the support function of research network staff), although some practices reported starting to look at sharing research resources and participation across practices as part of the primary care network,^20,21^ rather than as individual organisations. This could provide a platform for wider impact if benefits were more widely shared.

Formal ‘action and participatory research’ is less common in primary care and thus it is unsurprising that this was rarely reported. More generally, much of the research reported here represented general practice teams supporting ‘external’ research activity through recruitment and consenting of patients, rather than research initiated by practice teams, or reflecting a closer collaboration and partnership between research and practitioner communities.^22^ It is possible that these closer collaborations and participation are a much more impactful platform for change compared with the more traditional research that dominated most of the activity reported here.

The core mechanism we did identify was research activity affecting the job satisfaction of those members of staff who participate, where the break from routine activity, change of pace, and sense of achievement seemed to be salient for many staff. This is an interesting counterpoint to concerns that research activity is blocked by system pressures.^23^ It is not known whether this reflects the particular pressures on the general practice workforce,^24^ although such impacts have been identified in other professional groups outside medicine.^25^ It is also not clear if these impacts are specific to research or could be replicated by any alternative activity such as teaching or quality improvement work.^26^ Negative impacts on practice performance were rarely raised, although the levels of research activity in many practices was modest and issues of time and opportunity costs would have been less apparent.

### Implications for research and practice

The conclusion of a comprehensive review looking at the link between research activity and outcomes highlighted the need ‘*to build understanding of mechanisms, and to explore potentially negative impacts of research engagement alongside benefits’*.^9^ Our work suggests that a variety of mechanisms are plausible, although the effects of research activity on job satisfaction and practitioner wellbeing were most salient among our responders. We sought evidence of negative impacts, but responders reported few in principle and none in practice. Future research could usefully explore the differential impacts of different types of research, and whether the benefits reported here could be maximised through particular models of research activity or additional facilitation and support.

## Data

The datasets used and/or analysed during the current study are available from the corresponding author on reasonable request.
